# Recovery and prediction of postoperative muscle power – is it still a problem?

**DOI:** 10.1186/s12871-017-0402-7

**Published:** 2017-08-22

**Authors:** Martin Zoremba, Dennis Kornmann, Benjamin Vojnar, Rene Burchard, Thomas Wiesmann, Hinnerk Wulf, Thomas Kratz

**Affiliations:** 1 Department of Anaesthesia and Intensive Care Medicine, University of Marburg, Baldinger Strasse, D-35033 Marburg, Germany; 2 Department of Anaesthesia and Intensive Care Medicine, Clinique Bénigne Joly, 4 Allée Roger Renard, F-21240 Talant, France; 3 Department of Anaesthesia, Intensive Care Medicine and Pain therapy, Kreisklinikum, Weidenauerstrasse 76, D-57076 Siegen, Germany

**Keywords:** Muscle power, Nmba, Pulse oximetric saturation

## Abstract

**Background:**

In the postoperative period, immediate recovery of muscular power is essential for patient safety, but this can be affected by anaesthetic drugs, opioids and neuromuscular blocking agents (NMBA). In this cohort study, we evaluated anaesthetic and patient-related factors contributing to reduced postoperative muscle power and pulse oximetric saturation.

**Methods:**

We prospectively observed 615 patients scheduled for minor surgery. Premedication, general anaesthesia and respiratory settings were standardized according to standard operating procedures (SOP). If NMBAs were administered, neuromuscular monitoring was applied to establish a Train of four (TOF)-Ratio of >0.9 before extubation. After achieving a modified fast track score > 10 at 4 time points up to 2 h postoperatively, we measured pulse oximetric saturation and also static and dynamic muscle power, using a high precision digital force gauge. Loss of muscle power in relation to the individual preoperative baseline value was analysed in relation to patient and anaesthesia-related factors using the T-test, simple and multiple stepwise regression analysis.

**Results:**

Despite having achieved a TOF ratio of >0.9 a decrease in postoperative muscle power was detectable in most patients and correlated with reduced postoperative pulse oximetric saturation. Independent contributing factors were use of neuromuscular blocking agents (*p* < 0.001), female gender (*p* = 0.001), TIVA (*p* = 0.018) and duration of anaesthesia >120 min (*p* = 0.019).

**Conclusion:**

Significant loss of muscle power and reduced pulse oximetric saturation are often present despite a TOF-Ratio > 0.9. Gender differences are also significant. A modified fast track score > 10 failed to predict recovery of muscle power in most patients.

**Trial registration:**

German Clinical Trial Register DRKS-ID DRKS00006032; Registered: 2014/04/03

## Background

Use of neuromuscular blocking agents (NMBA) is associated with postoperative respiratory complications [1, 2]; they have a significant effect on neuromuscular recovery [3]. Even a small delay in recovery of pharyngeal muscular tone can induce upper airway collapse as well as micro-aspiration leading to respiratory dysfunction or pneumonia [4, 5]. Thus perioperative (quantitative) neuromuscular monitoring, in particular acceleromyography, is essential to ensure a TOF-Ratio > 0.9 before extubation [6, 7]. Although regarded as the gold standard, it may not be able to detect lesser degrees of neuromuscular block [8, 9]. Thus postoperative residual neuromuscular blockade (RNMB) cannot be completely excluded by the use of neuromuscular monitoring [10]. Even with intermediate duration of anaesthesia, it can occur after a single dose of NMBA [11]. Other anaesthesia- and patient-related factors can affect postoperative muscle strength and recovery [12–15]. The overall clinical relevance of postoperative muscle strength impairment within the postoperative period is frequently underestimated because the clinical signs of RNMB are not necessarily obvious [16]. Current standards for its detection in association with upper airway collapse have been developed in healthy subjects in the absence of other detrimental factors such as sedatives and analgesics [17, 18]. In this cohort study we evaluated the factors contributing to loss of muscle power in the immediate postoperative period after a TOF-ratio of ≥0.9 had been obtained.

## Methods

Ethics committee approval was obtained (University of Marburg, ref. no. AZ 176/09; German Clinical Trial Register DRKS-ID DRKS00006032). Informed consent was obtained in every patient included. As a result of the study design no written consent was demanded by the Ethics committee. We included 801 adult patients who were scheduled for minor surgery in general anaesthesia into this prospective observational study. They were nursed postoperatively in our post anaesthetic care unit (PACU) between 31st of january, 2010 and 31st of december 2012. In order to display daily routine no randomization was performed. Premedication and general anaesthesia was performed according to our standard operating procedures (SOP). Primary outcomes were defined as lack of postoperative muscle power and the identification of possible independent contributing risk factors. Secondary outcome is the impact of these factors on postoperative pulse oximetric saturation. Patients receiving neostigmine for reversal were excluded.

### Patients

Inclusion criteria were: age ≥ 18 years, American Society of Anaesthesiologists status (ASA) I-III, minor surgery), general anaesthesia. Minor surgery was defined as elective surgery, superficial surgery procedures, estimated blood loss <500 ml, modest postoperative pain expected, no prone positioning.

Excluded were patients of minor age (<18 years), and subjects undergoing thoracoabdominal surgery, as patients who were scheduled for surgery in regional anaesthesia.

### General anesthesia

Twelve hours before surgery, patients received clorazepate 20–40 mg orally. In the anaesthesia induction room, standard monitoring according to Association of Anaesthetists of Great Britain and Ireland (AAGBI) guidelines was established. General anaesthesia was induced according to our SOP using fentanyl 3 μg kg^−1^ and propofol 1.5–2.5 mg kg^−1^. A single dose of rocuronium (0.5 mg kg^−1^ ideal body weight) was given for tracheal intubation. When laryngeal mask was used, NMBA was avoided. No further neuromuscular blocking agent was given during surgery. All patients were ventilated to maintain an end-tidal CO_2_ pressure of approximately 4–4.7 kPa. A maximum peak pressure of 30 cmH_2_O was allowed for pressure-controlled ventilation with tidal volumes of 6–7 ml kg^−1^. The inspiration to expiration ratio was 1:1.5 and a positive end expiratory pressure of 5–8 cmH_2_O, 8–10 cmH_2_O when Body mass index (BMI) exceeded 35 kg cm^−2^ was applied throughout in all patients, using an adjusted FiO_2_ of 0.5 during anaesthesia maintenance and a FiO_2_ of 1.0 before extubation. To achieve comparable anaesthetic depths, self-adhesive Bispectral Index (BIS) electrode strips (BIS Quattro™; Aspect Medical Systems, Freising, Germany) were positioned on the forehead as recommended. General anaesthesia was maintained by sevoflurane (0.5–2 Vol%) or propofol infusion (6-12 mg kg^−1^) and intermittent bolus application of fentanyl 0.5–2 μg kg^−1^ to maintain BIS within a range of 35–60. According to our standard operating procedures the train of four (TOF) ratio (uncalibrated) was controlled (TOF-Watch™, Organon Teknika, Eppelheim Germany) to >0.90 before extubation ensured by a member of our research staff team (study nurse/postgraduate). A warming blanket device (Bair Hugger™, Arizant, Trittau, Germany) was applied during surgery. Each patient received dexamethasone (4 mg i.v., after induction) and dolasetron (25 mg i.v., 15 min before extubation) as prophylaxis against nausea and vomiting. When the patients were fully awake, alert and breathing spontaneously, the lungs were extubated on an inspiratory oxygen concentration of 100%. After transport to the PACU, they were nursed in the half sitting head up position for full recovery. Patients, reversed from residual neuromuscular blockade, were not included for further analysis.

### Evaluation of fast track score & postoperative management

Postoperative surveillance and nursing was according to our local guidelines. No sedatives were administered in the PACU. Research staff members observed and assessed the fast track score according to White and Song [19] after patient arrival at PACU at the fixed time points. PACU time was calculated when a score of 10 or more of 14 was achieved. All patients remained in the PACU until the measurements at T2 h were completed. Antiemetic drugs were given according to our local guidelines. VAS was evaluated at 15-min intervals. All patients received intravenous (i.v.) non-opioid analgesia (metamizole 15-25 mg kg^−1^) 15 min before the estimated end of surgery. In the PACU, visual analogue scale (VAS) scores were evaluated at 15-min intervals. Piritramide i.v. was given by the PACU nurses whenever the VAS exceeded 4. After PACU discharge, basic analgesia on the ward was performed as requested with ibuprofen and metamizole oral (nurse-controlled). Overall drug consumption as well as the related VAS scores were recorded within the first 24 hours.

### Muscle power measurements

As previously described static and dynamic hand muscle power was measured using a high precision digital force gauge (Force gauge FMI-100, Alluris, Freiburg, Germany) in a modified thumb pressure approach [20]. Baseline muscle power measurement was performed at the pre-anaesthetic visit after thorough demonstration of the method. We registered peak thumb pressure as the best of three attempts. In the PACU, measurements were performed as soon as the patient was alert and cooperative (Fast Track score > 10 (T0 h); pain and dyspnoea were assessed during coughing before and, if necessary, after analgesic therapy. All measurements were repeated in the PACU by research staff members at 30 min (T0.5 h), 1 h (T1 h) and 2 h (T2 h) after PACU arrival.

### Pulse oximetry

Pulse oximetry was standardized. It was measured on 3 different fingers ensuring a stable signal quality and using the best value without any supplemental oxygen. All included patients had a postoperative body temperature > 36° during PACU stay. Baseline pulse oximetry was performed at the pre-anaesthetic visit and postoperative measurements were made in the PACU by research staff members after a Fast Track score > 10 (T0 h) had been achieved at the same time points as above. Oxygen (1–6 L/min) was administered by face mask when pulse oximetric saturation (SpO_2_) values were <90%.

### Statistic analysis

In order to review all the potential risk factors for reduced postoperative pulse oximetric saturation and muscle power, a systematic review of the literature was performed using the search terms – (‘muscle power’ OR ‘reduced pulse oximetric saturation’ AND (‘predict’ OR ‘risk model’ OR ‘risk factor’) – and other combinations of these terms [1, 10, 11, 15, 21–26]. The following were all included for analysis against the variables postoperative muscle strength and pulse oximetric saturation, and their interaction; NMBA usage, age, gender, BMI, airway method (Larynx mask (LMA)/Intubation (ITN)), premedication, maintenance (balanced anaesthesia/total intravenous anaesthesia – TIVA), anaesthesia duration, neck circumference, postoperative opioid consumption, and fast track score > 10 within 20 min in PACU.

Postoperative SpO_2_ and muscle power were analysed as percentages of baseline. After dichotomization of the continuous data, univariate comparisons between the individual factors and target variables (SpO_2_/Muscle power) were calculated for each variable at each time point. The Kolmogorov-Smirnov t-test or Mann–Whitney U-tests for non-normally distributed variables was applied for continuous variables. Variables with a two-sided nominal *P* value of less than 0.2 in either analysis were further investigated jointly with a logistic regression framework. A stepwise mixed logistic regression analysis was used to develop the final prediction models for impaired muscle power or SpO_2_, respectively. Statistic analysis was realised with JMP 8 for MAC (SAS Institute Inc., Cary, NC).

## Results

Overall, 801 patients were included in our study; their basic biometric and specific perioperative anaesthesia related data was collected by study nurses. Patients receiving supplemental regional anaesthesia techniques were excluded. After thorough screening, 615 complete datasets were included for final analysis (Table 1). Overall cohort data exhibit except of TIVA vs balanced anaesthesia no differences in dichotomized data. Detailed evaluation of the main cohort data (+NMBA/−NMBA) indicate differences in distribution within the respective *t*-test an chi-square tests (Table 1).

**Table 1:** Basic cohort data including patients’ demographics, peri- and postoperative anaesthetic procedural data and medications (615 patients) T-test/chi-square evaluation of the main study populations (NMBA+/NMBA-)

	Cohort data (*n* = 615)	NMBA+ (*n* = 347)	NMBA– (*n* = 268)	*p*-Value
Gender (M/F)	278/337	185/162	93/175	0.001
Age	53 ± 15	55 ± 14	50 ± 16	0.001
BMI	27.5 ± 4.9	28 ± 5.0	26.4 ± 4.5	0.001
TIVA/Balanced Anaesthesia (n)	378/237	74/273	105/163	0.001
Premedication (Clorazepat mg)	27 ± 12	28 ± 11	26 ± 11	0.059
Fentanyl (mg)	0.44 ± 0.3	0.49 ± 0.2	0.36 ± 0.2	0.001
BIS intraoperative	39 ± 7	38 ± 6	39 ± 7	0.0141
BIS end	42 ± 11	42 ± 10	43 ± 11	0.174
Metamizole (mg)	1.7 ± 0.5	1.7 ± 0.5	1.6 ± 0.4	0.068
Piritramide (mg)	7.8 ± 4.5	7.9 ± 4.0	7.6 ± 5.0	0.480
Anaesthesia time (min)	130 ± 51	139 ± 43	117 ± 45	0.001
Fast Track score > 10 PACU (min)	25 ± 21	28 ± 23	22 ± 16	0.001
SpO_2_.pre-OP (%)	97.2 ± 1.5	97.0 ± 1.5	97.4 ± 1.4	0.002

*Abbreviations*: *NMBA* neuromuscular blocking agents, *M* male, *F* female, *BMI* body mass index, *TIVA* Total intravenous anaesthesia, *BIS* Bispectral Index, *PACU* Post anaesthetic care unit, *SpO*_*2*_ pulse oximetric saturation, *pre-OP* preoperative

Plus minus value: mean ± SD

### Postoperative muscle power

**Fig. 1:**
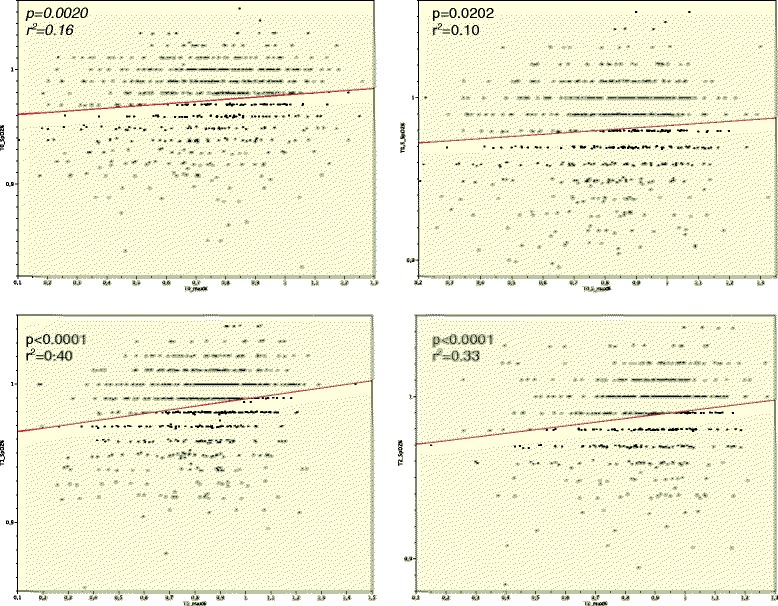
Muscle power/pulse oximetric saturation. Simple regression analysis: Correlation of pulse oximetric Saturation and muscle power in terms of NMBA usage (+/−) y-axis = pulse oximetric saturation (percentage of preoperative baseline) x-axis = peak muscle power (percentage of preoperative baseline)

Significant reduction of muscle power was detectable postoperatively in most patients (Table 2, Fig. 1). Within the univariate analysis, NMBA usage exhibit the strongest statistic effect on both muscle power and SpO_2_ at all time points (*p* < 0.0001). Other factors also exhibited significant effects on muscle power (Table 3). Loss of postoperative muscle power for patients receiving and not receiving NMBAs was affected by female gender and TIVA; NMBA usage increased the individual effect of these factors (Tables 4 and 5). Thus independent significant factors within a stepwise regression model at each measurement point were NMBA usage (*p* < 0.0001), female gender (*p* < 0.0001) and TIVA (*p* < 0.027). The Fast Track score failed to predict significant muscle power loss in most patients.

**Table 2:** Basic postoperative measurements of muscle power and SpO_2_ in PACU (*individual percentage of preoperative baseline*)

	T0 h	T0.5 h	T1 h	T2 h
*Muscle power (%) n = 615*	*74.3* ± *22*	*82.0* ± *20*	*82.4* ± *20*	*86.4* ± *19*
Muscle power + NMBA (%) *n* = 347	66.8 ± 21	77.2 ± 21	77.7 ± 20	84.0 ± 21
Muscle power –NMBA (%) *n* = 268	82.8 ± 21	87.1 ± 19	88.5 ± 18	89.6 ± 17
*SpO*_*2*_ *(%) n = 615*	*97.2* ± *3*	*98.0* ± *3*	*98.4* ± *3*	*98.8* ± *sd2*
*SpO*_*2*_ + NMBA (%) *n* = 347	96.7 ± 3	97.6 ± 3	98.1 ± 3	98.4 ± sd3
*SpO*_*2*_–NMBA (%) *n* = 268	97.9 ± 3	98.5 ± 2	99.0 ± 2	99.1 ± sd2

*Abbreviations*: *PACU* Post anaesthetic care unit, *SpO*_*2*_ pulse oximetric saturation, *NMBA* neuromuscular blocking agents

Plus minus value: mean ± SD, *p*-values displayed in Tables 3 and 6

**Table 3:** Effects on postoperative muscle power (t-Test, *P*-value)

	T0 h	T0.5 h	T1 h	T2 h
Gender (M/F)	0.0030	<0.0001	<0.0001	<0.0001
Age (≥55)	0.0412	n.s.	n.s.	n.s.
BMI (≥30)	n.s.	n.s.	n.s.	n.s.
NMBA (NMBA+/NMBA-)	<0.0001	<0.0001	<0.0001	<0.0001
TIVA/Balanced Anaesthesia	n.s.	0.0241	0.0177	0.0135
Clorazepate (≥20 mg)	0.0066	n.s.	n.s.	n.s.
Anaesthesia time(≥120 min)	0.0002	0.0115	0.0116	0.0073
Fast Track score in PACU (≥10 min.)	n.s.	0.0038	<0.0001	0.0016

*Abbreviations*: *NMBA* neuromuscular blocking agents, *M* male, *F* female, *BMI* body mass index, *TIVA* Total intravenous anaesthesia, *BIS* Bispectral Index, *PACU* Post anaesthetic care unit, muscle power throughout lower: male, NMBA+, short and long anaesthesia time (>120 min) partial effect visible towerds lower muscle power: TIVA

**Table 4:** NMBA usage and additional detrimental factors contributing to a lack in postoperative muscle power (t-Test, *p*-Value)

Muscle power	NMBA – (*n* = 268)				NMBA + (*n* = 347)			
	T0 h	T0.5 h	T1 h	T2 h	T0 h	T0.5 h	T1 h	T2 h
Gender (M/F)	0.0031	<0.0001	<0.0001	0.0012	<0.0001	<0.0001	<0.0001	<0.0001
Age (≥55)	n.s.	n.s.	0.0433	0.0056	n.s.	n.s.	0.0433	n.s.
BMI (≥30)	n.s.	n.s.	n.s.	n.s.	n.s	n.s.	n.s.	n.s.
TIVA/Balanced Anaesthesia	0.0153	0.0139	<0.0001	0.0014	0.0007	<0.0001	<0.0001	0.0038
Clorazepat (≥20 mg)	0.0210	n.s.	n.s.	n.s.	n.s.	n.s.	n.s.	n.s.
Anaesthesia time(>120 min)	n.s.	0.0389	n.s.	n.s.	0.0446	n.s.	n.s.	0.0231
Fast Track score/PACU(≥10 min.)	n.s.	n.s.	n.s.	n.s.	n.s.	0.0006	<0.0001	0.0046

*Abbreviations*: *NMBA* neuromuscular blocking agents, *M* male, *F* female, *BMI* body mass index, *TIVA* Total intravenous anaesthesia, *BIS* Bispectral Index, *PACU* Post anaesthetic care unit

**Table 5:** NMBA usage and additional detrimental factors contributing to a lack in postoperative SpO_2_ (t-Test, *p*-Value)

SpO_2_	NMBA – (*n* = 268)				NMBA + (*n* = 347)			
	T0 h	T0.5 h	T1 h	T2 h	T0 h	T0.5 h	T1 h	T2 h
Gender (M/F)	n.s.	n.s.	0.0067	0.0078	n.s.	n.s.	0.0274	0.0049
Age (≥55)	n.s.	n.s.	n.s.	0.0056	n.s.	n.s.	0.0433	n.s.
BMI (≥30)	n.s.	n.s.	n.s.	n.s.	n.s	n.s.	n.s.	n.s.
TIVA/Balanced Anaesthesia	n.s.	n.s.	n.s.	0.0014	0.0012	0.0002	0.0107	0.0488
Clorazepat (≥20 mg)	n.s.	n.s.	n.s.	n.s.	n.s.	n.s.	n.s.	n.s.
Anaesthesia time (≥120 min)	n.s.	n.s.	0.0121	0.0112	n.s.	n.s.	n.s.	n.s.
Fast trackFast track oreAldrete score in PACU (≥10)	n.s.	n.s.	n.s.	n.s.	n.s.	0.0404	0.0016	n.s.

*Abbreviations*: *NMBA* neuromuscular blocking agents, *M* male, *F* female, *BMI* body mass index, *TIVA* Total intravenous anaesthesia, *BIS* Bispectral Index, *PACU* Post anaesthetic care unit

### Postoperative pulse oximetric saturation

**Fig. 2:**
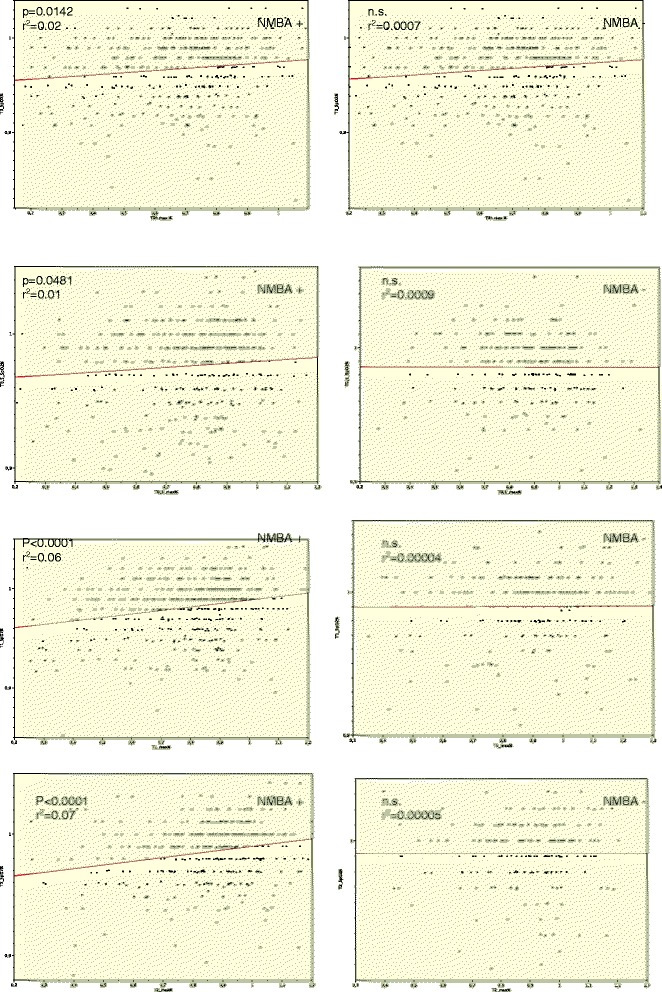
NMBA+ vs. NMBA −/muscle power and pulse oximetric saturation. Simple regression analysis: Correlation of pulse oximetric Saturation and muscle power in terms of NMBA usage (+/−) y-axis = pulse oximetric saturation (percentage of preoperative baseline) x-axis = peak muscle power (percentage of preoperative baseline)

Pulse oximetric saturation was decreased at all measurement points (Table 2). Within the univariate analysis, NMBA use had the most significant effect on postoperative SpO_2_; other factors also exerted effects (Table 6). Reduced postoperative muscle strength and decreased SpO_2_ indicates a certain correlation over the 2 h (Fig. 1). However, in patients not receiving NMBAs, there was no such correlation (Fig. 2).

**Table 6:** Effects on postoperative pulse oximetric saturation (t-Test, *P*-value)

	T0 h	T0.5 h	T1 h	T2 h
Gender (M/F)	n.s.	n.s.	0.0249	0.0033
Age (≥55)	n.s.	n.s.	n.s.	n.s.
BMI (≥30)	n.s.	n.s.	n.s.	n.s.
NMBA (NMBA+/NMBA-)	<0.0001	<0.0001	<0.0001	0.0013
TIVA/Balanced Anaesthesia	n.s.	n.s.	n.s.	n.s.
Clorazepat (≥20 mg)	n.s.	n.s.	n.s.	n.s.
Anaesthesia time(≥120 min)	0.0134	0.0157	0.0014	0.0070
Fast Track score in PACU (≥10 min.)	n.s.	n.s.	0.0019	0.0269

*Abbreviations*: *NMBA* neuromuscular blocking agents, *M* male, *F* female, *BMI* body mass index, *TIVA* Total intravenous anaesthesia, *BIS* Bispectral Index, *PACU* Post anaesthetic care unit

Pulse oximetric saturation troughout lower: NMBA+ short and long anaesthesia time (>120 min)

Partial effect visible towards lower pulse oximetric saturation: male, fast track score < 10

## Discussion

Fast track procedures in perioperative care have been established over the last decade even in high-risk patients. Quick recovery of muscular power is clearly essential for early mobilisation and overall outcome [27]. Safety standards as neuromuscular monitoring and standard operating procedures have led to a reduction in perioperative complications [28, 29]. Although we have maintained these safety standards, our data show a loss of postoperative muscle power after general anaesthesia, undetected by the Fast Track scoring in the PACU. There was also an associated reduced postoperative SpO_2_. Despite (uncalibrated) acceleromyographic neuromuscular monitoring (aNMM) being applied, ensuring a TOF Ratio ≥ 0.9 before extubation, use of NMBAs has the biggest impact on postoperative muscle power. Uncalibrated a NMM has a negative predictive value of 40%; i.e. we cannot predict recovery of muscle power in the majority of our study population. Calibration of the accelerometer as well as establishing a TOF Ratio of 1.0 before extubation improves the predictive value up to 97% (77% uncalibrated) [8] but at some time cost. Thus, the overall cost of treatment increases.

Additionally, longer surgery times after only a single dose of a neuromuscular blocking agent do not themselves prevent residual neuromuscular block [10].

Controversy exists concerning the antagonisation after NMBA usage as a standard procedure, e.g. using neostigmine as detrimental side effects including bradycardia and prolongation of the QT interval, nausea and vomiting, and an increase in bronchial tone can occur [30]. The use of cyclodextrines (Suggamadex®) is an effective alternative with some blockers, but overall anaesthesia costs increase [31].

Omitting NMBA by using alternative airway devices e.g. the laryngeal mask airway (LMA) has still its limitations and is not appropriate in every case [32]. Additionally a complete eschewal of NMBA for orotracheal intubation is impracticable and harmful [33, 34].

Other factors contributing to a lack of postoperative muscle power can be identified. We have shown clear gender differences, partly explained by the gender bias in accelerometry which is not usually adjusted for [35]. Although NMBAs were administered according to ideal body weight, the different fat/muscle/body water proportion in females may alter drug distribution [36]. Gender specific drug variation has already been confirmed for some anaesthesia related drugs [37, 38].

Hypnotic drugs can have similar effects on pharyngeal muscle tone. Volatile anaesthetics reinforce neuromuscular block at hypnotic, but not subhypnotic, concentrations [39]. Propofol however has a negative impact on pharyngeal tone and postoperative oxygenation even at subhypnotic concentrations [15, 40]. In contrast to the short term wash-out kinetics of most volatile anaesthetics, propofol has enduring effects on the basis of the specific elimination kinetics within the first 2 postoperative hours [41, 42], an effect enhanced with increasing age. An overall dosage reduction of propofol e.g. with supplemental remifentanil application seems reasonable.

Additionally, aging can possibly alter these effects mostly attributable to a decrease in hepatic metabolisation. This raises the question of whether current standards e.g. fast-track scores or TOF ratio of 0.9 before extubation are suitable for every patient. Under certain conditions these procedures fail or are inadequate. In particular, NMBA usage, female gender and TIVA can exhibit a potential effect in terms of muscle impairment resulting in an upper airway collapse, desaturation and contribute to major complications. In this regard our study setting

was not designed to reveal hard endpoints such as pneumonia, in-hospital mortality or myocardial infarction but rather to display possible interactions of both patient and anaesthesia related factors.

This could finally lead to an individualised risk stratification and anaesthesia management towards postoperative muscle power. In this context possible gender differences in both in pharmacodynamics and NMM as well as pharmacological interactions of TIVA and NMBA should be of further interest.

### Limitations

Anyhow, our study has some limitations. The cohort design has no active randomization displaying daily routine and were only single centre collected. Hence our statements can merely partially generalized. Although the complete dataset has a homogenous distribution, the two main subcohorts (+NMBA/−NMBA) within our study population exhibited statistic significant differences. Nevertheless, in general these differences were small and statistic significance was mostly attributable to the large study population rather than clinical relevance. Despite that, a cohort bias cannot be completely excluded.

NMM was standardized and verified by our study nurses before extubation. Nevertheless this method has a potential bias. Additionally we applied an uncalibrated semiquantitative NMM which can exhibit a greater bias rather than a calibrated device.

Muscle power tests using the hand pressure device were potentially influenced by both patient vigilance and study nurse performance. In order to minimize interindividual bias we performed all measurements as a best of three attempt and analysed only the best attempt (at every time point) displayed as percentage of preoperative baseline measurement in each patient.

Our study was intended to identify possible risk factors rather than find major morbidity in minor surgery patients. We cannot completely exclude further drug interactions, as we did not measure plasma concentrations of the respective NMBA or hypnotics. Finally our findings may not be representative due to the single centre analysis but could be a starting point for future investigation of the interaction of single factors.

## Conclusion

A TOF-Ratio of >0.9 does not prevent reasonable muscle power loss within the perioperative period which has a significant negative impact on pulsoximetric saturation. Additional independent detrimental factors were female gender and TIVA. Thus an overall reduction in NMBA usage even as a single dosage is reasonable.

## Abbreviations

NMBA: Neuromuscular blocking agents
NMM: Neuromuscular monitoring
PACU: Post anaesthesia care unit
RNMB: Residual neuromuscular blockade
SOP: Standard operating procedure
TIVA: Total intravenous anaesthesia
TOF: Train of four
